# High syphilis incidence among PrEP‐adherent men who have sex with men and transgender women in Peru

**DOI:** 10.1002/jia2.70002

**Published:** 2025-08-27

**Authors:** Silver K. Vargas, Kelika A. Konda, Ronaldo I. Moreira, Iuri C. Leite, Marcelo Cunha, Brenda Hoagland, Juan V. Guanira, Marcos Benedetti, Cristina Pimenta, Beatriz Grinztejn, Valdiléa G. Veloso, Carlos F. Caceres

**Affiliations:** ^1^ Centro de Investigación Interdisciplinaria en Sexualidad SIDA y Sociedad Universidad Peruana Cayetano Heredia Lima Peru; ^2^ Fundação Oswaldo Cruz (INI‐Fiocruz) Instituto Nacional de Infectologia Evandro Chagas Rio de Janeiro Brazil

**Keywords:** key populations, MSM, PrEP adherence, PrEP, syphilis, transgender women

## Abstract

**Introduction:**

Syphilis remains a public health concern in Peru. Pre‐exposure prophylaxis (PrEP) implementation programmes in Latin America need to assess their impact on sexually transmitted infections (STIs), along with their feasibility. We assessed the relationship between PrEP adherence and syphilis incidence among men who have sex with men (MSM) and transgender women (TW) enrolled in ImPrEP, a multi‐country PrEP demonstration project; however, this analysis focuses on Peru.

**Methods:**

Between April 2018 and June 2021, 2292 HIV‐negative MSM/TW attending Peruvian STI clinics were enrolled and followed in ImPrEP. Participants had to be aged ≥18 years and report recent condomless anal sex (CAS), sex with a partner living with HIV, STI history (diagnosis/symptoms) and/or transactional sex. Quarterly follow‐up visits included PrEP dispensing, behavioural assessment, HIV and syphilis screening (treponemal test and Rapid Plasma Reagin [RPR] if syphilis negative at enrolment; RPR only if reactive‐treponemal test at baseline). PrEP adherence was assessed using the medication possession ratio (MPR: #pills prescribed / #days between visits). Generalized estimating equation (GEE) Poisson regression models were used to evaluate factors related to syphilis incidence and also assessed syphilis incidence during two periods: pre‐COVID‐19 lockdown (up to 16 March 2020) and during COVID‐19‐lockdown (17 March 2020−June 2021).

**Results:**

We enrolled 2039 cisgender‐MSM and 253 TW, with a median follow‐up time of 514 days; 205 incident syphilis cases were identified among 185 individuals. Overall syphilis incidence was 9.1 cases/100 person‐years (p.y.) (95% CI: 7.9−10.4), 14.7/100 p.y. (95% CI: 10.5−20.1) among TW and 8.3/100 p.y (95% CI: 7.1−10.0) among cisgender‐MSM. During the COVID‐19 pre‐lockdown period, syphilis incidence was 10.0/100 p.y. (95% CI: 8.3−12.1) and 8.1/100 p.y. (95% CI: 6.6−10.0) during‐lockdown. Multivariate GEE analysis showed higher syphilis incidence among PrEP‐adherent participants (MPR≥0.6) (adjusted incidence rate ratio [aIRR]: 1.46 [95% CI: 1.08−1.99]), those reporting receptive CAS (aIRR: 1.53 [95% CI: 1.11−2.11]) and TW (aIRR: 1.64 [95% CI: 1.08−2.51]). Syphilis incidence pre‐lockdown was higher for participants reporting receptive CAS (aIRR: 2.35 [95% CI: 1.43−3.86]); during‐lockdown, syphilis incidence was higher among those diagnosed with syphilis at enrolment (aIRR: 2.70 [95% CI: 1.67−4.36]).

**Conclusions:**

Syphilis incidence is high among PrEP‐adherent MSM/TW, those reporting receptive‐CAS and among TW. Health systems implementing PrEP should strengthen existing STI prevention strategies and incorporate new ones, like Doxy‐PEP for PrEP‐adherent MSM, TW and individuals engaging in receptive‐CAS. MPR may be a tool to identify PrEP users at risk for syphilis.

## 1 INTRODUCTION

Syphilis is a re‐emerging infection with 7.1 million new cases worldwide in 2020 [1]. In Latin America, men who have sex with men (MSM) and transgender women (TW) have the highest syphilis prevalence [2]. In Peru, syphilis prevalence is 0.5% among the general population [3]; 13.8% in MSM/TW in coastal and jungle cities [4], and 22% among TW in Lima [5]. Key risk factors include condomless anal sex (CAS), multiple sex partners and HIV [6, 7, 8, 9].

Pre‐exposure prophylaxis (PrEP) is a proven strategy for HIV prevention [10, 11, 12]. PrEP efficacy is closely linked to adherence, required to achieve and maintain protective drug concentrations [13]. PrEP adherence can be monitored by directly measuring drug levels in biological samples [14] or by indirect monitoring like pill counts, self‐reported use and medication possession ratio (MPR) [15].

PrEP studies have reported mixed results regarding sexually transmitted infections (STIs) incidence and sexual behaviour [16, 17, 18, 19]. PROUD reported decreased risk‐reduction strategies like condom use and serosorting after PrEP initiation [20]. High STI frequency among PrEP users was attributed to “risk compensation,” where PrEP's protection against HIV encouraged sexual risk behaviours, increasing STI vulnerability [21, 22]. Evidence suggests high STI acquisition among MSM during PrEP use, accompanied by decreased condom use [23, 24] and high PrEP adherence [25]. Among Australian MSM, STI rates remained stable during PrEP use compared to before‐PrEP periods [26], and Dutch MSM/TW PrEP users had high STI incidence, which remained stable throughout follow‐up despite declines in sex partners and CAS [27]. Researchers identified among Belgian MSM/TW a relationship between recurrent anal sex and high STI incidence, with high PrEP adherence [28]. Among Californian MSM PrEP users, those with the lowest drug concentrations had higher syphilis rates compared to individuals with optimal drug concentrations (≥4 doses/week) who engaged in CAS with multiple partners [29]. Additionally, poor PrEP adherence is frequent among key populations like TW and young MSM [30]. Studies suggest an unclear relationship between PrEP use and STI frequency. While PrEP users manage their HIV risk through PrEP, they remain at risk for STIs, including syphilis.

Using PrEP adherence as a proxy for retention in care and STI risk assessment—rather than relying solely on reported sexual behaviours—may help identify MSM/TW PrEP user subgroups with varying syphilis risk. This approach could provide valuable evidence for the implementation and/or development of targeted STI control strategies within PrEP implementation programmes in Latin America. We evaluated the association of PrEP adherence and syphilis incidence among MSM/TW participants in ImPrEP Peru. ImPrEP was conducted in Brazil, Mexico and Peru from 2018 to 2021, to assess the feasibility of daily oral PrEP delivery for MSM and TW within public health settings [31]. Given that ImPrEP coincided with the COVID‐19 pandemic, which affected overall healthcare provision [32], we assessed syphilis incidence across two time periods divided by the lockdown declaration date in Peru.

## 2 METHODS

### 2.1 Study design and population

This secondary analysis used longitudinal data from the ImPrEP study, a prospective, single‐arm, open‐label, multicentre study that evaluated same‐day oral PrEP implementation in Brazil, Mexico and Peru. Inclusion criteria were HIV‐negative MSM and TW, aged ≥18 years reporting one or more of the following behaviours 6 months prior to enrolment: CAS, anal sex with HIV‐positive partner(s), STI symptoms/diagnosis or transactional sex [31]. Only data from Peru, collected between May 2018 and June 2021, were included to evaluate the association between PrEP adherence and syphilis incidence.

### 2.2 Study measures and procedures

Participants were recruited through social media, healthcare providers and peer‐educator referrals. MSM/TW attending 10 STI clinics—five in the capital, Lima, four in coastal cities and one in the eastern rainforest—were invited to participate while seeking PrEP or HIV/STI testing services. After providing informed consent, participants underwent an eligibility assessment, including HIV testing. Eligible individuals who tested HIV negative received PrEP. Demographics, sexual behaviour and substance use data were collected at enrolment and quarterly visits. Baseline information included age, gender, reason to attend the STI clinic, binge drinking (≥five drinks in a 2‐hour period), STI symptoms (genital rash, lesions/warts, anal/urethral discharge), STI diagnosis (syphilis/chlamydia/gonorrhoea), sex with a partner living with HIV, stimulant use and transactional sex. Longitudinal information on sexual behaviour (receptive CAS and number of sex partners), STI (syphilis/chlamydia/gonorrhoea) testing and PrEP‐adherence assessment information were collected at quarterly follow‐ups and included in data analysis. PrEP adherence was measured using MPR, defined as the ratio of pills dispensed over days between visits. Participants with an MPR ≥0.6 (equivalent to taking ≥ 4 weekly pills, enough to reach 96% protective efficacy) were considered adherent [13, 15].

### 2.3 Syphilis assessment

Syphilis screening was performed at baseline and all follow‐ups using the Determine Syphilis treponemal test (Abbott, France) and the Rapid Plasma Reagin (RPR) test (Wiener Lab, Argentina), with serum obtained from centrifuged venous blood. Incident syphilis cases were defined as occurring during follow‐up through treponemal and RPR seroconversion or a ≥4‐fold increase in RPR titres compared to the previous RPR result after treatment for the prior syphilis episode. Free‐of‐charge treatment was provided to all syphilis cases, according to Peruvian guidelines [33].

### 2.4 Statistical processing and analysis

Descriptive analysis of baseline characteristics included percentages, medians and interquartile ranges (IQRs). PrEP adherence (MPR) was calculated for all participants with at least one follow‐up visit, then dichotomized using 0.6 as the cut‐off for multivariate analysis. Syphilis incidence was analysed using generalized estimating equations (GEE) with Poisson regression including demographic and sexual behaviour variables. For individuals who tested negative for syphilis at enrolment, time‐at‐risk began at enrolment. For those diagnosed with active syphilis at enrolment, time‐at‐risk started upon achieving serological cure (defined as a four‐fold RPR titre decline from baseline). Multiple syphilis episodes were considered by tracking RPR titre variation. GEE models facilitate the evaluation of multiple events, accounting for correlations between repeated measures per participant, including time‐varying variables such as PrEP adherence, receptive CAS and number of sex partners.

Additionally, we assessed the impact of the COVID‐19 emergency on syphilis incidence by generating a model for different time periods, prior to lockdown on 16 March 2020 (pre‐lockdown) and from 17 March 2020 to June 2021 (during‐lockdown). Syphilis incidence rates were calculated as the number of incident cases per person‐years of follow‐up, estimated for the entire cohort and stratified by PrEP adherence, gender and time period (pre‐ and during‐lockdown).

This secondary analysis was approved by the Ethics Committee of Universidad Peruana Cayetano Heredia (SIDISI 200930). Data confidentiality was maintained following legal and institutional guidelines.

## 3 RESULTS

Between May 2018 and June 2021, 2292 participants enrolled in the ImPrEP demonstration study in Peru and received PrEP. The median age was 27 years (IQR: 23−33) and 253 (11.0%) were TW. Among participants, 63.1% sought PrEP, while 36.9% were enrolled when accessing other services (e.g. HIV/STI testing, HIV post‐exposure prophylaxis, etc.), 6.1% reported stimulant use and 73.7% reported binge drinking. At enrolment, 59.1% reported receptive CAS, the median number of sex partners was 4 (IQR: 2−10), 57.2% were unaware of partners’ HIV status and 10.3% reported CAS with a partner living with HIV. Additionally, 22.4% reported engaging in transactional sex; 20.2% had STI symptoms/diagnoses; and 9.6% had active syphilis (RPR≥1:8) at enrolment (Table 1). During follow‐up, 1603 individuals had at least one quarterly visit; the median follow‐up time was 514 days (IQR: 227−752), with a median of 5 visits (IQR: 3−7); median MPR for all visits was 0.92 (IQR: 0.69−0.99). The proportion of participants reporting receptive CAS during follow‐up ranged from 56.3% to 64.7%, while the proportion of those reporting ≤4 partners ranged from 57.3% to 60.4% (data not shown).

**Table 1:** Baseline characteristics among participants from Peru of ImPrEP project

Characteristics	Total 2292 (100%)	MSM 2039 (88.9%)	TW 253 (11.1%)
Age (years)^a^	27 (23−33)	27 (23−34)	33 (25−39)
18−24	805 (35.1)	745 (36.5)	60 (23.7)
25−34	977 (42.6)	877 (43.0)	100 (39.5)
>35	510 (22.2)	417 (20.5)	93 (36.8)
Stimulant use^b^			
Yes	140 (6.1)	105 (5.2)	35 (13.8)
No	2152 (93.9)	1934 (94.9)	218 (86.2)
Binge drinking^b^			
Yes	1678 (73.2)	1480 (72.6)	198 (78.3)
No	614 (26.8)	559 (27.4)	55 (21.7)
PrEP adherence (MPR)^a^, ^b^	0.92 (0.69−0.99)	0.92 (0.71−1)	0.90 (0.65−0.99)
Active syphilis diagnosis^d^			
Yes	220 (9.6)	190 (9.3)	30 (11.9)
No	2073 (90.4)	1849 (90.7)	223 (88.1)
Receptive condomless anal sex			
Yes	1355 (59.1)	1126 (55.2)	229 (90.5)
No	937 (40.9)	913 (44.8)	24 (9.5)
Number of sexual partners^a^, ^c^	4 (2−10)	3 (2−7)	16 (4−50)
≤4	1153 (50.3)	1093 (53.6)	60 (23.7)
5−10	608 (26.5)	564 (27.7)	44 (17.4)
≥ 11	532 (23.2)	382 (18.7)	149 (58.9)
Sex with HIV‐infected partners			
No	744 (32.5)	662 (32.5)	82 (32.4)
Yes	236 (10.3)	229 (11.2)	7 (2.8)
Unknown status of partner	1312 (57.2)	1148 (56.3)	164 (64.8)
Transactional sex			
Yes	513 (22.4)	336 (16.5)	177 (70.0)
No	1779 (77.6)	1703 (83.5)	76 (30.0)
Recent STI diagnosis or symptoms			
Yes	463 (20.2)	403 (19.8)	60 (23.7)
No	1829 (79.8)	1636 (80.2)	193 (76.3)
Reason for visiting STI clinic			
Looking for PrEP	1448 (63.2)	1353 (66.4)	95 (37.6)
Other (PEP, HIV testing)	844 (36.8)	686 (33.6)	158 (62.4)

^a^ Median (p25−p75).

^b^ Last 3 months.

^c^ Corresponds to all follow‐up visits.

^d^ Active syphilis case was defined with RPR≥1:8.

Overall syphilis incidence was 9.1 cases/100 person‐years (p.y.) (95% CI: 7.9−10.4), corresponding to 205 cases among 185 individuals. Among PrEP adherents (MPR≥0.6), syphilis incidence was 10.0/100 p.y. (95% CI: 8.4−11.7), whereas among non‐adherents (MPR<0.6), it was 7.4/100 p.y (95% CI: 5.6−9.5). Syphilis incidence was 14.7/100 p.y. (95% CI: 10.5−20.1) among TW and 8.3/100 p.y (95% CI: 7.1−9.7) among MSM. Syphilis incidence pre‐lockdown was 10.0/100 p.y. (95% CI: 8.3−12.1), and 8.1/100 p.y. (95% CI: 6.6−10.0) during‐lockdown.

**Figure 1:**
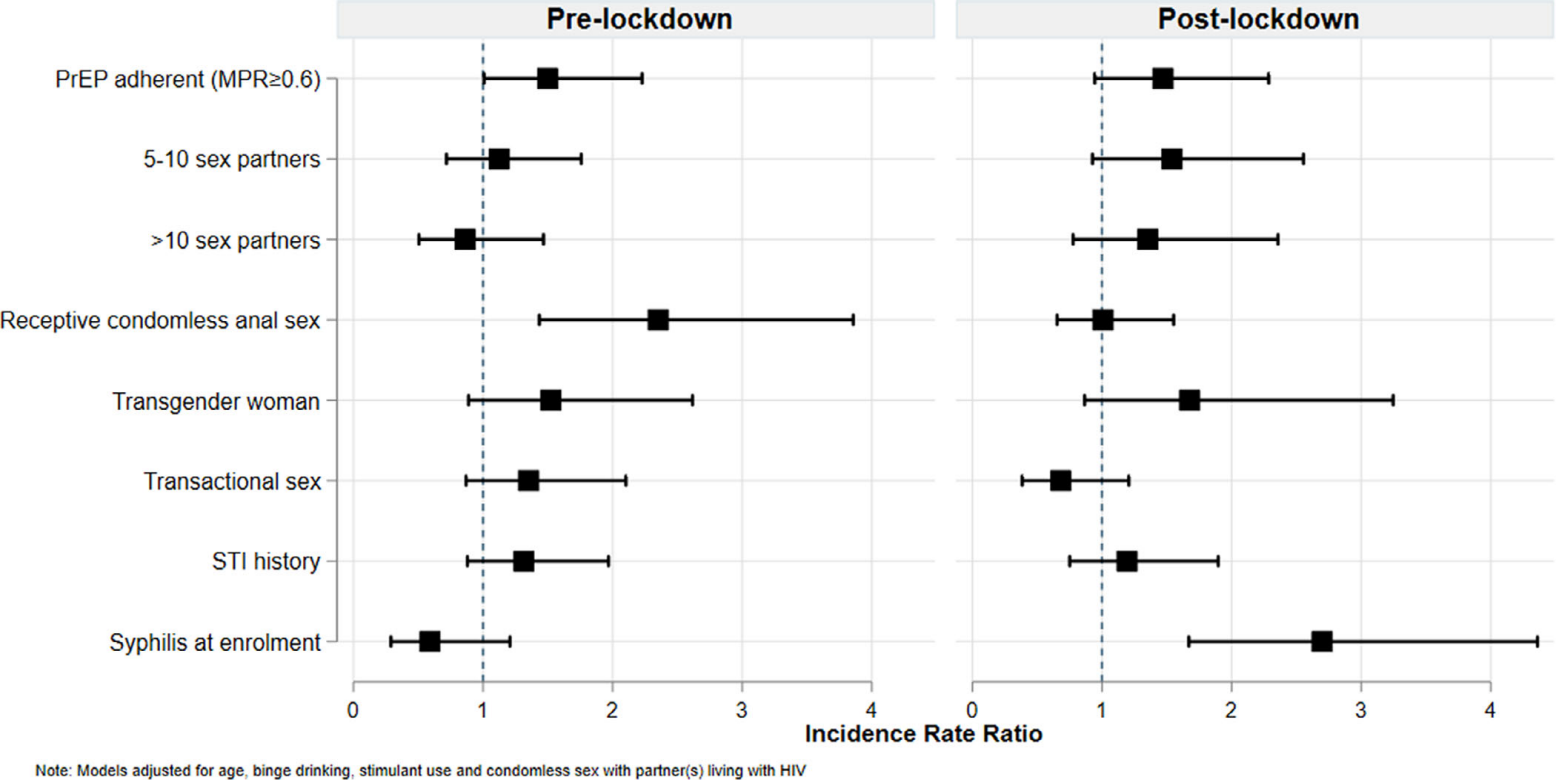
**Factors associated with syphilis incidence before and during lockdown due to the COVID‐19 health emergency**.

Multivariate GEE analysis shows higher syphilis incidence among PrEP adherents (adjusted incidence rate ratio [aIRR]: 1.46 [95% CI: 1.08−1.99]), participants reporting receptive CAS (aIRR: 1.53 [95% CI: 1.11−2.11]) and TW (aIRR: 1.64 [95% CI: 1.08−2.51]); however, participants ≥35 years had lower syphilis incidence (aIRR: 0.65 [95% CI: 0.44−0.96]) than those aged 18–24 (Table 2). Pre‐lockdown, syphilis incidence was higher among PrEP adherents (aIRR: 1.50 [95% CI: 1.01−2.23]) and participants reporting receptive CAS (aIRR: 2.35 [95% CI: 1.43−3.86]). During‐lockdown, syphilis incidence was higher among those diagnosed with syphilis at enrolment (aIRR: 2.70 [95% CI: 1.67−4.36]) (Figure 1).

**Table 2:** Factors associated with syphilis incidence among participants from Peru of ImPrEP project

	Bivariate analysis			Adjusted model		
Characteristics	IRR	CI 95%	*p* value	IRRa	CI 95%	*p* value
Gender^a^						
Cis	Ref.			Ref.		
Trans	**1.81**	**1.27**−**2.57**	**0.001**	**1.66**	**1.09**−**2.53**	**0.021**
Age^a^						
18−24	Ref.			Ref.		
25−34	0.97	0.71−1.34	0.861	0.96	0.70−1.32	0.811
≥35	0.73	0.50−1.04	0.086	**0.65**	**0.44**−**0.96**	**0.028**
Stimulant use^a^, ^b^						
No	Ref.			Ref.		
Yes	1.05	0.50−2.20	0.898	0.92	0.44−1.95	0.828
Binge drinking^a^, ^b^						
No	Ref.			Ref.		
Yes	**1.40**	**1.03**−**1.89**	**0.031**	1.32	0.97−1.80	0.077
PrEP adherence						
MPR <0.6	Ref.			Ref.		
MPR ≥0.6	1.34	0.99−1.80	0.055	**1.46**	**1.08–1.99**	**0.013**
Active syphilis diagnosis^a^, ^b^, ^c^						
No	Ref.			Ref.		
Yes	**1.50**	**1.02**−**2.22**	**0.040**	1.36	0.91−2.04	0.135
Receptive condomless anal sex^b^						
No	Ref.			Ref.		
Yes	**1.66**	**1.23**−**2.27**	**0.001**	**1.53**	**1.11**−**2.11**	**0.009**
N° sexual partners^b^						
≤4	Ref.			Ref.		
5−10	1.34	0.97−1.85	0.072	1.29	0.92−1.80	0.138
≥ 11	1.36	0.96−1.94	0.088	1.02	0.69−1.54	0.893
Sex with HIV‐infected partners^a^						
No	Ref.			Ref.		
Yes	0.92	0.57−1.49	0.741	1.03	0.63−1.68	0.893
Unknown status of partner	1.15	0.85−1.57	0.356	1.11	0.81−1.53	0.514
Recent STI diagnosis or symptoms^a^						
No	Ref.			Ref.		
Yes	1.34	0.99−1.81	0.059	1.23	0.90−1.69	0.190
Transactional sex^a^						
No	Ref.			Ref.		
Yes	**1.36**	**1.00**−**1.85**	**0.031**	1.04	0.74−1.46	0.831

*Note*: Bold values indicate statistical significance.

Abbreviations: IRR, incidence rate ratio; IRRa, adjusted incidence rate ratio.

^a^ Corresponds to enrolment visit.

^b^ Last 3 months.

^c^ Active syphilis case was defined with RPR≥1:8.

## 4 DISCUSSION

In our study, we assessed the relationship between PrEP adherence and syphilis incidence among MSM and TW enrolled in the multi‐country PrEP demonstration project ImPrEP, and found that syphilis incidence is higher among PrEP‐adherent MSM/TW. Our findings align with others showing that STI incidence, including syphilis, is associated with PrEP adherence among MSM/TW. Among highly PrEP adherent (4−7 pills/week) MSM and TW from California, high syphilis incidence was reported as compared to those with low PrEP adherence (<4 pills/week), although not statistically significant [34]. A retrospective U.S. PrEP study showed higher STI incidence during periods of PrEP use compared to before PrEP use, with the highest STI incidence among the most PrEP‐adherent individuals [35]. Higher STI incidence was observed among daily PrEP users compared to event‐driven PrEP users [36, 37]. Additionally, in Alabama, STI incidence was higher among highly PrEP‐adherent black MSM who reported multiple sex partners [38]. Syphilis incidence is high among PrEP‐adherent MSM/TW, who are eligible for PrEP due to sexual behaviours such as CAS or multiple sex partners; these behaviours may persist during PrEP use, continuing their risk for syphilis and other STIs. PrEP adherence including clinic visits for additional medication ensures regular STI testing, facilitating prompt detection and treatment. Furthermore, assessing PrEP adherence using MPR could help identify MSM/TW subgroups with varying syphilis risk, as PrEP‐adherent users may be at high syphilis risk, requiring sexual behaviour assessment, STI screening and treatment or being offered additional STI prevention interventions. Whereas individuals with poor PrEP adherence may require interventions to improve retention and HIV testing due to potential low drug concentration.

We found high syphilis incidence among participants reporting receptive CAS and among TW. Clark et al. reported higher active syphilis (RPR≥1:8) prevalence among MSM reporting receptive sex (15.1%), compared to insertive sex (0.6%) [39]. Dutch MSM PrEP users reported increased rates of receptive CAS and anal STIs compared to pre‐PrEP periods and non‐PrEP users [40]. Among Canadian MSM, CAS was a mediator between PrEP use and bacterial STIs [41]. In Lima, TW had a higher treponemal test prevalence compared to cisgender MSM [42] and higher active syphilis [4]. In our study, TW reported higher rates of receptive CAS and transactional sex than MSM. TW face significant barriers to care, underscoring the need for targeted interventions given their higher vulnerability to syphilis and HIV [43, 44]. Regular STI screening, prevention counselling and non‐condom‐based STI prevention strategies like partner notification [45] and expedited partner therapy [46] are crucial interventions for STI control in Peru. Post‐exposure doxycycline prophylaxis [47] reduces the incidence of *C. trachomatis* and syphilis among MSM/TW PrEP users; targeting higher‐risk subgroups is essential to mitigate the risk of antimicrobial resistance [48].

Overall, syphilis incidence was 9.1/100 p.y. higher than the 7.3/100 p.y. reported in the IPrEX trial [49] and an Australian open‐label PrEP implementation study (8.0/100 p.y.) [50], but close to a meta‐analysis’ pooled estimated syphilis incidence of 9.5/100 p.y. among MSM PrEP users [51]. Data from a multi‐country survey showed that self‐reported syphilis among MSM almost doubled between 2010 and 2017, with PrEP use as one key factor for this increase, while the number of non‐stable partners and increased STI testing mediated the relationship between PrEP use and syphilis diagnosis [52].

The COVID‐19 pandemic disrupted HIV/STI services, reducing case detection during lockdown [53, 54, 55]. Syphilis incidence was higher among those reporting receptive CAS before lockdown, and might be explained because this time period accumulated more follow‐up visits, and factors associated with syphilis incidence would behave similarly to the model using whole cohort data. Syphilis diagnosis at enrolment was associated with high syphilis incidence during lockdown, likely because risk behaviours persist regardless of PrEP use. Retention among TW, already low before the pandemic, worsened due to pandemic‐related barriers [30, 56]. Poor PrEP adherence among TW is often attributed to a lack of information and inadequate PrEP implementation [57, 58], exacerbated by the COVID‐19 emergency [59, 60], with TW disproportionately losing jobs and facing limited healthcare access [61].

Our study limitations include the syphilis case definition relied on laboratory data, and potentially missing early syphilis infections with non‐reactive RPR tests or treponemal rapid tests [62], though all participants underwent a clinical exam. Such cases are rare and would not significantly affect findings. PrEP adherence was measured using MPR, an indirect method that correlates well with blood drug levels [15]. Direct drug concentration in biological samples is the gold standard for PrEP‐adherence measures but unsuitable for public PrEP programmes. Early loss to follow‐up was high among TW and young adults, both at higher HIV risk [63, 64], but exhibiting lower PrEP retention [65, 66, 67], which may potentially biased follow‐up. The COVID‐19 lockdown measures impaired retention for several months; however, biosafety protocols for re‐opening facilitated the resumption of follow‐up in ImPrEP sites. Despite these limitations, our study provides important insights into syphilis dynamics among MSM and TW using PrEP in Peru. PrEP improves sexual health and may not change long‐term risk behaviours [68], but provides an opportunity to integrate and deliver strategies to help PrEP users identify and manage their syphilis risk [69].

## 5 CONCLUSIONS

We found that syphilis incidence was higher among PrEP‐adherent MSM and TW, and among those engaging in receptive CAS. MPR may serve as a tool to identify individuals at syphilis risk, and PrEP programmes require strengthening current STI prevention measures or introducing new strategies for PrEP users at risk for syphilis.

## COMPETING INTERESTS

All authors report no potential competing interests.

## AUTHORS’ CONTRIBUTIONS

VGV, CFC and BG conceived and designed the ImPrEP study. SKV performed the statistical analyses, interpreted the findings and drafted the manuscript. KAK supervised the current analysis and manuscript preparation. RIM, ICL and MC supervised the statistical analyses. BH, JVG, MB and CP were involved in revising the manuscript for important intellectual content. All authors read and approved the final manuscript.

## FUNDING

The ImPREP study was made possible thanks to Unitaid's funding and support. Unitaid is a hosted partnership of WHO. This article was partially prepared as part of the activities of the Doctoral Program in Epidemiologic Research offered by Universidad Peruana Cayetano Heredia (UPCH) funded by the Training Grant EF033‐235‐2015 FONDECYT/CIENCIACTIVA and part of the Grant D43 TW007393 “Peru Infectious Diseases Epidemiology Research Training Consortium,” sponsored by the Fogarty International Center of the US National Institutes of Health (NIH/FIC). The funding bodies had no role in the design of the study and collection, analysis and interpretation of data and in writing the manuscript.

## Data Availability

Data will be available upon reasonable request and will be approved by the ImPrEP coordination team.
